# Supplementary material for: Novel Loss-of-Function Variants in CDC14A are Associated with Recessive Sensorineural Hearing Loss in Iranian and Pakistani Patients
Source: Int J Mol Sci. 2020 Jan 2;21(1):311. doi: 10.3390/ijms21010311 (PMC6982189; doi:10.3390/ijms21010311)
Supplement: Supplementary file 1 [file ijms-21-00311-s001.pdf]

Supplementary Table S1: List of analyzed HL associated genes and their corresponding OMIM numbers.

Gene	OMIM number
ABHD12	613599
ACTG1	102560
ADCY1	103072
ADGRV1	602851
AIFM1	300169
ARSG	610008
ATP1A3	182350
ATP2B2	108733
BDP1	607012
BSND	606412
BTD	609019
CABP2	607314
CCDC50	611051
CD164	603356
CDC14A	603504
CDH23	605516
CEACAM16	614591
CEP250	609689
CEP78	617110
CHD7	608892
CHST3	603799
CIB2	605564
CISD2	611507
CLDN14	605608
CLIC5	607293
CLPP	601119
CLRN1	606397
COCH	603196
COL11A1	120280
COL11A2	120290
COL2A1	120140
COL4A3	120070
COL4A4	120131
COL4A5	303630
COL4A6	303631
COL9A1	120210
COL9A2	120260
COL9A3	120270
CRYM	123740
DCDC2	605755
DIABLO	605219
DIAPH1	602121
DIAPH3	614567
DMXL2	612186
EDN3	131242
EDNRB	131244
ELMOD3	615427

EPS8	600206
EPS8L2	614988
ERAL1	607435
ESPN	606351
ESRP1	612959
ESRRB	602167
EYA1	601653
EYA4	603550
FAM65B	611410
FGF3	164950
FGFR1	136350
FGFR2	176943
FITM2	612029
FOXI1	601093
GAB1	604439
GATA3	131320
GIPC3	608792
GJB2	121011
GJB3	603324
GJB6	604418
GPSM2	609245
GRHL2	608576
GRXCR1	613283
GRXCR2	615762
GSDME	608798
HARS	142810
HARS2	600783
HGF	142409
HOMER2	604799
HSD17B4	601860
IFNLR1	607404
ILDR1	609739
KARS	601421
KCNE1	176261
KCNJ10	602208
KCNQ1	607542
KCNQ4	603537
KIAA0391	609947
KIT	164920
KITLG	184745
KMT2D	602113
LARS2	604544
LHFPL5	609427
LMX1A	600298
LOXHD1	613072
LOXL3	607163
LRP5	603506
LRTOMT	612414
MARVELD2	610572
MASP1	600521

MCM2	116945
MET	164860
METTL13	617987
MIR96	611606
MITF	156845
MPZL2	604873
MSRB3	613719
MYH14	608568
MYH9	160775
MYO15A	602666
MYO3A	606808
MYO6	600970
MYO7A	276903
NARS2	612803
NISCH	615507
OSBPL2	606731
OTOA	607038
OTOF	603681
OTOG	604487
OTOGL	614925
P2RX2	600844
PAX3	606597
PCARE	613425
PCDH15	605514
PDZD7	612971
PEX1	602136
PEX6	601498
PEX7	601757
PHYH	602026
PJVK	610219
PNPT1	610316
POLD1	174761
POU3F4	300039
POU4F3	602460
PRPS1	311850
PTPN11	176876
PTPRQ	603317
RDX	179410
REST	600571
ROR1	602336
S1PR2	605111
SERPINB6	173321
SGO2	612425
SIX1	601205
SIX5	600963
SLC17A8	607557
SLC22A4	604190
SLC26A4	605646
SLC26A5	604943
SLC44A4	606107

SLC7A8	604235
SMPX	300226
SNAI2	602150
SOX10	602229
STRC	606440
SYNE4	615535
TBC1D24	613577
TBX1	602054
TECTA	602574
TIMM8A	300356
TJP2	607709
TMC1	606706
TMEM132E	616178
TMIE	607237
TMPRSS3	605511
TMTC2	615856
TNC	187380
TPRN	613354
TRIOBP	609761
TSPEAR	612920
TWNK	606075
USH1C	605242
USH1G	607696
USH2A	608400
WBP2	606962
WFS1	606201
WHRN	607928

Supplementary Table S2: Extended statistical information for quantification of RT-qPCR expression values in Figure 3.

Pairwise comparison	Exon 2-3	Exon 11-12
Control 1 – Control 2	n.s.	n.s.
Control 1 – Control 3	$p < 0.05$	n.s.
Control 1 – I.2	$p < 0.001$	$p < 0.001$
Control 1 – II.2	$p < 0.001$	$p < 0.001$
Control 2 – Control 3	n.s.	n.s.
Control 2 – I.2	$p < 0.01$	$p < 0.001$
Control 2 – II.2	$p < 0.001$	$p < 0.001$
Control 3 – I.2	$p < 0.05$	$p < 0.001$
Control 3 – II.2	$p < 0.001$	$p < 0.001$
I.2 – II.2	$p < 0.001$	$p < 0.01$

Results of post-hoc pairwise comparisons with Bonferroni´s correction for multiple comparisons are shown for exon 2-3 and exon 11-12.

Abbreviations: n.s. = not significant
